# Assessment of spinal cord motion as a new diagnostic MRI-parameter in cervical spinal canal stenosis: study protocol on a prospective longitudinal trial

**DOI:** 10.1186/s13018-019-1381-9

**Published:** 2019-10-12

**Authors:** Katharina Wolf, Axel J. Krafft, Karl Egger, Jan-Helge Klingler, Ulrich Hubbe, Marco Reisert, Marc Hohenhaus

**Affiliations:** 1 Department of Neurology and Neurophysiology, Faculty of Medicine, University of Freiburg, Breisacher Straße 64, 79106 Freiburg, Germany; 2 Department of Radiology, Medical Physics, Faculty of Medicine, University of Freiburg, Freiburg, Germany; 3 Department of Neuroradiology, Faculty of Medicine, University of Freiburg, Freiburg, Germany; 4 Department of Neurosurgery, Faculty of Medicine, University of Freiburg, Freiburg, Germany

**Keywords:** Phase-contrast MRI, Spinal cord, Spinal canal stenosis, Spinal cord compression, Degenerative cervical myelopathy (DCM), Spinal cord motion, CSF-flow

## Abstract

**Background:**

Increased spinal cord motion has been proven to be a relevant finding within spinal canal stenosis disclosed by phase-contrast MRI (PC-MRI). Adapted PC-MRI is a suitable and reliable method within the well deliberated setting. As the decision between conservative and operative treatment can be challenging in some cases, further diagnostic marker would facilitate the diagnostic process. We hypothesize that increased spinal cord motion will correlate to clinical course and functional impairment and will contribute as a new diagnostic marker.

**Methods:**

A monocentric, prospective longitudinal observational trial on cervical spinal canal stenosis will be conducted at the University Medical Center Freiburg. Patients (*n* = 130) with relevant cervical spinal canal stenosis, being defined by at least contact to the spinal cord, will be included. Also, we will examine a control group of healthy volunteers (*n* = 20) as proof-of-principle. We will observe two openly assigned branches of participants undergoing conservative and surgical decompressive treatment (based on current German Guidelines) over a time course of 12 month, including a total of 4 visits. We will conduct a broad assessment of clinical parameters, standard scores and gradings, electrophysiological measurements, standard MRI, and adapted functional PC-MRI of spinal cord motion. Primary endpoint is the evaluation of an expected negative correlation of absolute spinal cord displacement to clinical impairment. Secondary endpoints are the evaluation of positive correlation of increased absolute spinal cord displacement to prolonged evoked potentials, prediction of clinical course by absolute spinal cord displacement, and demonstration of normalized spinal cord motion after decompressive surgery.

**Discussion:**

With the use of adapted, non-invasive PC-MRI as a quantitative method for assessment of spinal cord motion, further objective diagnostic information can be gained, that might improve the therapeutic decision-making process. This study will offer the needed data in order to establish PC-MRI on spinal cord motion within the diagnostic work-up of patients suffering from spinal canal stenosis.

**Trial registration:**

German Clinical Trials Register, ID: DRKS00012962, Register date 2018/01/17

## Background

Despite immense clinical experience within the field of cervical spinal canal stenosis, in some cases the clinical decision-making process is still difficult. A definite indication for surgical treatment is given in case of present typical clinical symptoms in combination with electrophysiological sings of cord deterioration and conventional MRI showing spinal cord compression. But difficulties can occur in case of overlapping comorbidities, especially in older patients who are most commonly affected [1]. Additionally, an early and reliable detection of patients at risk to develop clinical myelopathy in advance of irreversible damage to the spinal cord would be desirable. At the same time, risk of overtreating “preventive” surgery based on the sole conventional MRI diagnosis of a relevant spinal canal stenosis needs to be avoided.

Phase-contrast MRI (PC-MRI) allows for a non-invasive quantitative assessment of motion, without application of contrast agents [2–4]. PC-MRI measurements use ECG-triggering and provide time-resolved data over the course of a heartbeat. The time-resolved velocity information can therefore be used to derive further quantitative parameters such as absolute displacement, stroke volume, and flow rate [5, 6]. A well-known uncertainty of PC-MRI data depends on the selection of the velocity encoding parameter (venc) that should be chosen according to the expected velocity values [7]. Phase-wraps, so-called aliasing, occur if the actual tissue velocity is higher than the venc.

Analysis of dynamic changes within the spinal canal or the aqueduct applying PC-MRI are currently gaining interest within different areas of research, focusing mainly on alterations of cerebrospinal fluid (CSF)-flow facing difficulties of the MRI-techniques due to partial volume error within the narrow CSF-space (e.g., [8–10]). Few studies so far have focused on the dynamics of the central nervous system (CNS), e.g., spinal cord motion [10–12]. It was demonstrated by application of PC-MRI that dynamic alterations within the spinal canal also contribute to the mechanical stress on the spinal cord [10–12]. A local increase of spinal cord motion at level of stenosis pointed towards a local stretch-phenomenon [10]. The increase in spinal cord motion related to functional impairment [10] and impaired sensory evoked potentials (SEP) within patients [11], which might indicate future diagnostic value of this objective quantitative dynamic parameter.

PC-MRI of the spinal cord generates functional information in addition to standard anatomical MRI. The assessment of spinal cord motion via adapted PC-MRI is a feasible and highly reliable imaging technique in contrast to CSF-flow analysis applying the same method [10].

## Methods

### Aim of the study

The results of this study should provide a new and reliable diagnostic tool in the field of cervical spinal canal stenosis by the use of adapted PC-MRI. The additional dynamic data of spinal cord motion should be demonstrated to be clinically relevant.

Primary endpoint is the evaluation of the expected negative correlation of absolute spinal cord displacement to clinical impairment. Secondary endpoints are assessments of expected positive correlation of increased absolute spinal cord displacement to prolonged evoked potentials, prediction of clinical course in spinal canal stenosis by absolute spinal cord displacement, demonstration of reduced spinal cord motion after decompressive surgery and comparison of standard MRI-techniques with PC-MRI.

### Design

This study is an investigator initiated prospective, longitudinal, monocentric observational trial. The trial consists of two openly assigned treatment branches: conservative treatment and surgical decompressive treatment.

The study protocol has been approved by the institutional ethics committee of the University of Freiburg, Germany (reference number 261/17). The trial was registered at the German Clinical Trials Register (DRKS00012962) on January 17, 2018. The study-population is linked to another trial at our center (MIDICAM trial). The screening period started in April 2018.

### Study population

We will analyze patients with monosegmental cervical spinal canal stenosis due to disc herniation or relevant degenerative spinal canal stenosis (*n* = 130). A second proof-of-principle control group, age- and gender-matched, of healthy participants will be included (*n* = 20).

### Inclusion and exclusion criteria

Patients, aged 18–90 years, with relevant cervical spinal canal stenosis due to disc herniation or degenerative spinal canal stenosis and written informed consent will be included. A relevant cervical spinal canal stenosis is defined as contact to the spinal cord with diminished CSF-space in the anterior or posterior compartments diagnosed in T2-weighted MRI. The spinal canal stenosis must be at a singular level only. Multisegmental relevant spinal cord compression will be excluded. Other exclusion criteria are patients with any contraindication for MRI, previous major operations of the cervical spine as well as non-mechanic causes of spinal canal stenosis or myelopathy (e.g., tumors, inflammation/infections, trauma) and other clinically relevant peripheral or central nervous system disease (e.g., multiple sclerosis, polyneuropathies).

Controls, aged 18–90 years and written informed consent given, are required to have no spinal canal stenosis and no impairments in daily living by other comorbidities at all. Also, general MRI contraindications will lead to exclusion from the study.

### Time-line and study protocol

**Fig. 1:**
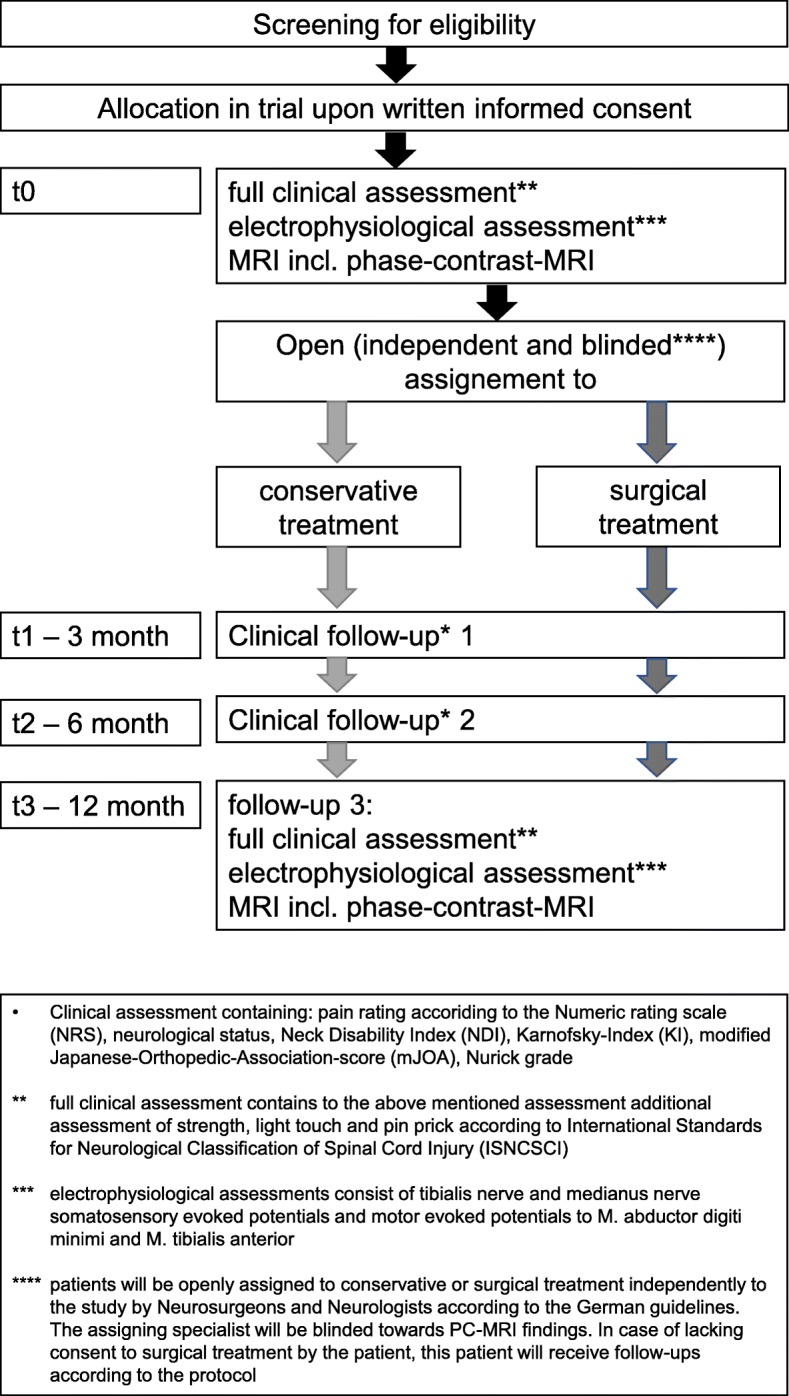
Study protocol

After enrolment within the study and primary visit (t0, including standardized neurological and electrophysiological assessments) patients will be assigned to conservative or surgical treatment (Fig. 1) based on German Guidelines [13]. The assignment will be conducted by study-independent neurosurgeons and neurologists of the University Medical Center Freiburg who are blinded to the PC-MRI results but will consider clinical and electrophysiological findings of the first visit. In case of not given consent to recommended surgery, patients will still receive follow-up visits according to the study protocol but will be indexed as assigned to the surgical treatment branch and statistically analyzed as a subgroup. All patients will receive clinical follow-up visits at 3 (t1), 6 (t2), and 12 (t3) months. At the primary visit (t0) and at 12 month (t3), all patients will undergo MRI assessments, expanded clinical assessments, and additional electrophysiological diagnostics.

### Clinical parameters

The epidemiological parameters (age, sex, onset of symptoms) are assessed at the primary visit (t0).

The following clinical parameters are assessed at each visit (t0–t3): the pain localization and intensity in combination with the limitations of the daily life are documented according to the numeric rating scale (NRS), the Neck Disability Index (NDI), and Karnofsky-Index (KI) (Fig. 1). The state of painkiller intake is documented regarding to the WHO classification. The severity of symptoms is rated by the modified Japanese-Orthopedic-Association-score (mJOA) [14] and Nurick grade [15]. Reflex status with three grades (attenuated, regular, enhanced) will be scored at upper and lower extremities with the site indicated. Also, a full standard neurological assessment will be conducted.

At t0 and t3, an additional assessment according to the International Standards for Neurological Classification of Spinal Cord Injury (ISNCSCI) [16] will be added.

All assessments will be performed by specialists of the Departments of Neurosurgery and Neurology. They will be blinded towards the results of PC-MRI.

### Electrophysiological parameters

Patients receive an electrophysiological workup at t0 and t3. The following standard measurements will be applied: somatosensory evoked potentials (SSEP) of the tibial and median nerve (including lumbar-, Erb-, and C2-interpeak-latencies, respectively), motor-evoked potentials (MEP) with additional nuchal and lumbar single-pulse to the M. tibialis anterior and the M. abductor digiti minimi [17]. The approximated central motoric latency is calculated by cortical MEP minus nuchal or lumbar MEP, respectively [17]. The results of the evoked potentials are also divided into four grades: normal [3], mildly deteriorated [2], severely deteriorated [1], and abolished [0], adapted to Kuhn et al. [18] and Petersen et al. [19]. Per individual, the need of further assessments to exclude other peripheral or central neurological comorbidities or to quantify additional radicular involvement is evaluated and indicated by an independent neurologist.

### MRI measurements and parameters

MRI measurements are performed using a 3 Tesla MRI scanner (SIEMENS Magnetom Prisma®). A standard T2-weighted (T2w) 3D sequence in sagittal orientation is acquired with the following parameters: spatial resolution 0.6 mm × 0.6 mm × 1.0 mm, TR 1500 ms, TE 134 ms, Flip angle 105°, GRAPPA factor: 3, acquisition time 3:53 min.

The evaluation of the spinal canal diameter is done in the sagittal and reformatted transverse T2w images (www.nora.de). Two independent raters will subjectively rate the presence of a T2w hyperintense myelopathy sign (yes/no). Additionally, the T2w signal intensity ratio (SIR) of the spinal cord at level of spinal canal stenosis and at the non-affected level C2 will be measured.

**Fig. 2:**
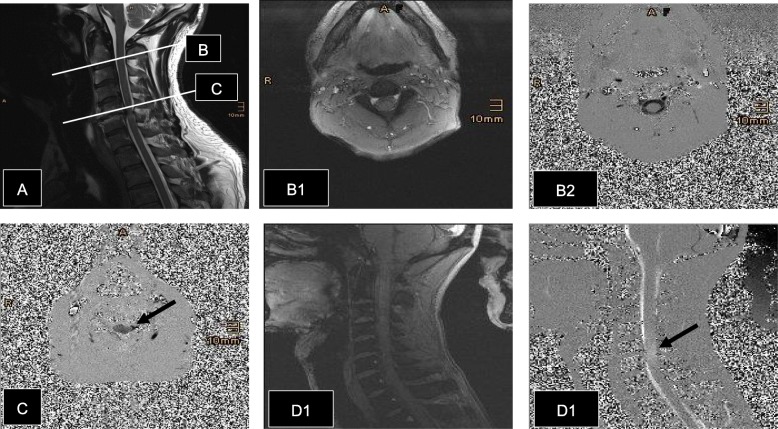
Patient with spinal cord compression at cervical level C5/6. **A** Sagittal T2w MRI, lines indicating axial levels C2 and C5. **B1** Axial magnitude. **B2** Axial phase-imaging at level C2, spinal cord and CSF-space can be is easily distinguished. The gray-level within the phase-images encode the measured velocities in cranio-caudal direction, dark gray velocities in caudal direction, light vice versa. Bright white or black are aliasing effect. The gray intensity signal of the spinal cord is similar to the surrounding neck tissues. **C** Axial phase-imaging at level of stenosis C5; there is barely any CSF-space left, only minor CSF-motion can be seen at the left side of the spinal cord (arrow). The gray intensity signal of the spinal cord is much darker than the surrounding neck tissues. **D1** Sagittal magnitude. **D2** Sagittal phase-imaging. Here, light gray signals encode cranio-caudal velocities and dark gray encodes the opposite direction. At the level of stenosis, a local increase in spinal cord motion can be observed (arrow)

Within a second step, the imaging protocol comprises the acquisition of the cardiac-gated (prospective ECG-triggering) 2D phase-contrast MRI data of the entire cervical spinal canal during free, steady breathing. To capture spinal cord motion which mainly occurs along the cranio-caudal direction, the direction of velocity encoding was set accordingly depending on the orientation of the PC-MRI images (Fig. 2) with a velocity-encoding parameter adapted to the expected velocities of spinal cord motion. PC-MRI data will be acquired in axial orientation at level of C2 and of stenosis with the following parameters: spatial resolution 0.9 mm × 0.9 mm × 5 mm, FoV 200 × 200 mm^2^, TR = 20.2 ms, TE = 7.7 ms, flip angle 15°, bandwidth 488 Hz/Pixel, venc 5 cm/s, PEAK-GRAPPA acceleration [20], acquisition time approximately 1.5 min depending on the heart rate. Within the phase-images velocities are optically encoded within a spectrum of gray; dark gray indicating motion in cranio-caudal direction, light gray vice versa (Fig. 2). In addition, PC-MRI data are acquired in sagittal orientation covering vertebra C2 to T1 is conducted with the following parameters: spatial resolution 1 mm × 1 mm × 3 mm, FoV 200 × 200 mm^2^, TR = 31.8 ms, TE = 7.75 ms, flip angle 15°, bandwidth 488 Hz/Pixel, venc 5 cm/s, PEAK-GRAPPA, acquisition time approximately 2 min depending on the heart rate. In sagittal phase-images, motion in cranio-caudal direction is encoded in light gray (Fig. 2).

The time-resolved velocity information of the PC-MRI measurements is further used to calculate the absolute displacement per segment and its cranio-caudal ratio via calculation of the area under the velocity-time curve. We will not include velocity amplitudes or maximum velocities into statistical analysis to avoid group differences due to interindividual variation of the heart rate that influences the spinal dynamics as discussed in Wolf et al. [10].

### Statistical analysis

The sensitivity of conventional T2w imaging is reported at 60% [21, 22]. In order to improve the diagnostic sensitivity to at least 80%, the group size calculation was based on an aimed power of 90% assuming a type I error of 5%, reaching a sample size of *n* = 114 patients.

Statistics are performed by SPSS IBM Statistics®, *p* < 0.05 will be considered statistically significant. The validity of all subjective and processed MRI measurements will be calculated using intra-class correlation coefficients (ICCs, single measures, two-way mixed effects model). The ICC values will be characterized as follows: “fair” for 0.41 to 0.6, “moderate” for 0.61 to 0.8, and “substantial” for 0.81 to 1 [23]. ICC ≤ 0.6 will not be considered acceptable.

For comparison between means, we will perform Rang-Test for independent samples. The correlation and prediction value of pathological clinical and electrophysiological factors with dynamic alterations will be calculated through multiple regression analysis. As there is no generally accepted definition of myelopathy in current literature and guidelines, we defined “functional cervical myelopathy” as presence of a relevant spinal canal stenosis in combination with mJOA-score ≤ 15 in combination with an at least mildly deteriorated SSEP of the Median nerve. By classification of our patients into two groups (functional present or abstinent myelopathy), we will be able to determine the sensitivity and specificity of the new imaging procedure.

## Discussion

In cervical spinal canal stenosis, the deterioration of the spinal cord is caused by several mechanisms related to loss of blood-supply leading to neurotoxic effects beginning within the central gray matter and later affecting the entire spinal cord segment [24]. Mostly, these mechanisms are attributed to the increasing pressure on the spinal cord and the subarachnoid vessels [24].

With application of PC-MRI, oscillations of the spinal cord in healthy conditions have been noted within several studies (e.g., [25–27]). In physiological conditions, the origin of spinal cord motion could be attributed to pulsatile impact of local arteries [28, 29] as well as effects related to breathing and brain movements, that itself derives from arterial in- and venous outflow [27]. The data of a pre-study indicated a positive correlation of spinal cord motion to age within healthy conditions but needs further evaluation [10]. Spinal cord motion in the non-comprised cervical spinal canal was at about 0.5–0.6 cm/s; intraindividually, there were only minor differences of spinal cord motion between cranio-caudal segments (index C2/C5 about 1) (e.g., [10]).

Within cervical spinal canal stenosis, three studies independently demonstrated that spinal cord motion at level of stenosis is significantly increased patients. Interestingly, the intraindividual difference of the total displacement (=area under the curve, or absolute spinal cord motion) at level of stenosis C5 was significantly increased compared to the intraindividual spinal cord motion at C2 up to a twofold or higher, pointing towards a local dynamic impact on the spinal cord [10]. Interrater-reliability of this method was higher than 90%, the method itself uses conventional PC-MRI sequences and is timely feasible [10].

Focusing on the dynamics of the spinal cord comprises two benefits in contrast to a so far mostly favored analysis of CSF-alterations: first, the affected CNS and its mechanical stress is much more likely to be directly linked to functional impairments other than being an epiphenomenon. Second, the spinal cord resembles a solid structure that can be easily assessed without any short distance alterations requiring tremendously high spatial resolution MRI and therefore a more complex and time-consuming acquisitions.

It has been discussed, whether the observed increased spinal cord motion measured by PC-MRI is an artifact of interstitial cell fluids or CSF-flow [12]. We do not share this assumption due to two facts: First, the spinal cord can be delineated from surrounding CSF (see also Fig. 2) so that a systematic error of the measured spinal cord velocities because of effects from CSF flow can be minimized by careful selection of a region of interest. This is supported by a high interrater-reliability as demonstrated with the pre-study [10]. Second, the measured mean velocity and associated total displacement values of up to 5–6 mm per heart beat within cranio-caudal direction over a region of almost 20 mm^2^ [10] are substantially larger as would be expected because of interstitial fluid flows.

This will be the first longitudinal clinical study on spinal cord motion in cervical spinal canal stenosis and its diagnostic value on functional impairment and clinical course.

### Limitations

Limits of the study are non-randomized design and in parts non-blinded analysis and acquisition of the data. Still, in order to demonstrate the objectivity of this analysis, we will provide sufficient proof of interrater-reliability by analysis of the MRI-datasets by two independent researchers.

## Data Availability

The datasets used and/or analyzed during the current study are available from the corresponding author on reasonable request.

## Abbreviations

CNS: Central nervous system
CSF: Cerebrospinal fluid
DCM: Degenerative cervical myelopathy
ISNCSCI: International Standards for Neurological Classification of Spinal Cord Injury
KI: Karnofsky Index
MEP: Motoric evoked potentials
mJOA: Modified Japanese-Orthopedic-Association-score
MRI: Magnet resonance imaging
PC-MRI: Phase-contrast MRI
SIR: Signal intensity ratio
SSEP: Somatosensory-evoked potentials
venc: Velocity encoding factor
